# Mechanism of action of glucocorticoids in nasal polyposis

**DOI:** 10.1016/S1808-8694(15)31101-0

**Published:** 2015-10-19

**Authors:** Atílio Maximino Fernandes, Fabiana Cardoso Pereira Valera, Wilma T. Anselmo-Lima

**Affiliations:** 1 Doctor, USP/Ribeirão Preto, Otorhinolaryngologist; 2 Doctor, Professor; 3 Livre-docente habilitation, Professor

**Keywords:** cytokines, glucocorticoids, pathophysiological mechanisms, nasal polyposis

## Abstract

Glucocorticoids (GC) are the drugs of choice for the clinical treatment of nasal polyposis, according to the medical literature. Its mechanism of action in the regression of clinical symptoms and polyps, however, is not fully understood. The topical and/or systemic use of glucocorticoids lead to variable expression of cytokines, chemokines and lymphokines, as well as changes in cells. It is known that GC suppresses the expression of pro-inflammatory cytokines, chemokines and adhesion molecules such as ICAM-1 and E-selectin; GC also stimulate the transcription of anti-inflammatory cytokines such as TGF-β. GC suppress pro-fibrotic cytokines related to polyp growth, such as IL-11, the basic fibroblast growth factor (b-FGF), and the vascular endotelial growth factor (VEGF). The action of GC depends fundamentally on their interaction with receptors (GR); certain subjects have a degree of resistance to its effect, which appears to be related with the presence of a β isoform of GR. GC also act variably on the genes involved in immunoglobulin production, presentation, and antigen processing.

**Aim:**

We present a review of the literature on the mechanisms of GC action in nasal polyosis.

**Conclusion:**

Understanding the mechanism of action of GC in nasal polyposis will aid in the development of new, more efficient, drugs.

## INTRODUCTION

Glucocorticoids (GC) are steroid hormones derived from cholesterol metabolism. These hormones are effective immunosuppressant and anti-inflammatory agents, and are naturally produced in the adrenal glands upon stimulation by the hypothalamic-pituitary-adrenal axis (HPA). These steroid hormones are the main therapeutic component in various autoimmune diseases such as rheumatoid arthritis, ulcerative colitis, asthma and polyposis.^1^

The fist successful use of hydrocortisone (the first GC metabolite that was synthesized) was in 1948; it was used for suppressing the clinical manifestations of rheumatoid arthritis. GC are used in nasosinal polyposis (NSP) in its topical or systemic forms, yielding excellent results by causing polypoid tissues to regress and by reducing inflammation. Clinical improvement occurs, with decreased respiratory obstruction and rhinorrhea, and improved olfaction. GC are first line drugs for the initial treatment of this condition, according to the International Consensus on NSP in 2002^2^ and the European Consensus in 2004;^3^ it's efficacy has been demonstrated in various therapeutic models.^4^^,^^5^^,^^6^ Topical GC are preferable for long-term use due to minimal systemic absorption and fewer side-effects; their effect within the paranasal sinuses, however, is limited. GC have also been used routinely following polyp-removing surgical procedures for reducing the recurrence of polyps and increasing the interval between surgery in these patients, thus helping control NSP.^6^ GC may, however, not be effective in all cases; the success rate of this form of treatment is around 60.9 to 80%.^7^^,^^8^ It is known that unsuccessful topical GC treatment is related to extensive NSP cases and lack of adhesion to therapy. The main factor that establishes the efficacy of GC therapy is the intrinsic, cell mechanism-mediated, resistance to the drug.

The aim of this review was to discuss the possible cell action mechanisms of GC in NSP and their interactions with cytokines, chemokines and adhesion molecules.

## REVIEW OF THE LITERATURE

### Molecular Mechanisms in NSP

NSP is a chronic inflammatory disease; its pathophysiological mechanisms have not been fully understood, notwithstanding recognition of the histological changes that occur. These alterations are epithelial hyperplasia, increased goblet cell, basal membrane thickening, stromal edema, and increased fibroblast production, to which is added inflammatory cell recruitment including lymphocytes, plasmocytes and particularly eosinophils.^6^

At a molecular level, cytokines - essential regulating proteins for immune cell growth, differentiation and activation - influence NSP by two mechanisms: local tissue growth stimulation mediated by, for instance, b-FGF, IGF-1, TGF-α, and TGF- β, and cytokine-mediated long-term maintenance of an inflammatory state involving IL-1β, TNF-α, IL-4, IL-5, IL-6, IL-10, and IFN-γ, among others.^9^ The complex interaction between these cytokines and structural and inflammatory cells leads to a chronic process that, after initiated, appears not to depend on the causal stimulus for its perpetuation.

It is known that fibroblasts and epithelial cells, once stimulated, are induced to produce TNFα and IL-1β. These cytokines stimulate nasal mucosa structural cells to produce various other cytokines (GM-CSF, IL-2, IL-3, IL-5, IL-6, IL-8, IL-12, IL-13, IL-16, TGF-α, TGF-β, TNF-α and IL-1 β), chemokines (RANTES, eotaxin, eotaxin-2) and adhesion molecules (ICAM-1 and VCAM-1). Cytokines activate inflammation, while chemokines and adhesion molecules are powerful circulating inflammatory cell -particularly eosinophils - recruiters. Once translocated to target tissues, these cells stimulate the production of other cytokines, which prolong and increase inflammation and produce cytotoxic molecules, such as the major basic protein (MBP), the eosinophilic cationic protein (ECP), leukotrienes and the platelet-activating factor (PAF). These molecules in turn intensify tissue damage, basal membrane thinning, stromal fibrosis, angiogenesis, and gland and stromal hyperplasia.^6^^,^^10^ Eosinophils are particularly able to produce cytokines - such as IL-3, IL-5 and GM-CSF - that increase their survival and that recruit additional eosinophils into the polyps, thus intensifying tissue eosinophilia.^11^

### GC Action on Cell Composition and Activation

The anti-inflammatory action of GC results from a number of changes in inflammatory cells. Their use leads to an increase in the number of circulating neutrophils, decreased production of inflammatory lymphokines and monokines - such as IL-1 and TNF-α11 - and induction of eosinophils apoptosis.

Mastruzzo et al.^12^ found that intranasal GC increase the density of CD8+ cells in polyps, thereby decreasing the CD4/CD8 ratio. This author also observed decreased numbers of IL-5+ and IL-4+ cells, in contrast with an increased number of TGF-β+ cells, which was correlated with CD8+ T-cells, and which is anti-inflammatory.^12^

### GC Intracellular action and on Inflammatory Mediators

The action mechanism of these substances on cells results from their bonding to cytoplasmic high-affinity glucocorticoid receptors (GR). The steroid-receptor complex (GC-GR) is capable of nuclear translocation; in the nucleus, it bonds with chromosomal DNA. This bond sets in motion or inhibits gene transcription, respectively by transactivation and transrepression. Transactivation is mediated by hormone-activated glucocorticoid receptor (or GC-GR complex) bonding to the DNA sequence named glucocorticoid response element (GRE). Corticosteroids, for instance, may stimulate anti-inflammatory molecule (IL-10, IL-1Rα6, TGF-β and IκB) and uteroglobulin synthesis.

Benson^9^ used the microarray technique on glucocorticoid-treated polyps and showed that 200 of 22,000 genes underwent some change in expression after treatment, and that these alterations were involved in various immunoglobulin-producing steps, in antigen presentation and processing, in chemoattraction, and in granulocyte and lymphocyte activation. The most highly expressed genes were those producing uteroglobulin and 15-lipoxigenase, the latter being an enzyme that induces lipoxin production (signaling enzymes for inhibiting inflammation). Uteroglobulin inhibits leukocyte chemotaxis and phospholipase A2 and pro-inflammatory cytokines synthesis.^9^

It is thought that most of the side effects of oral GC take place by gene transactivation. The main anti-inflammatory effect of GC, however, is related to their transrepressor effect. Transrepression is specially mediated by direct protein-protein inhibition in the GC-GR complex and by transcription factors such as AP-1 and NF-kB (cross-inhibition). When bonding with these factors, the GC-GR complex blocks the transcription of cytokines, chemokines and enzymes such as IL-18, IL-16 and eotaxin, being thus directly related to their expresion.^13^

Transcription factors are cytoplasmatic molecules that upon activation are able to translocate to the nucleus and to induce transcription of pro-inflammatory cytokines, adhesion molecules and chemokines, among others. GC, therefore, also suppress the transcription of pro-inflammatory cytokines (IL-1, IL-2, IL-3, IL-4, IL-, IL-6, GM-CSF, TNF-α, and IFN-γ), of chemokines (RANTES, eotaxin and MCP) and adhesion molecules (ICAM-1 and E-selectin).

Marx et al.^14^ found decreased GM-CSF and TNF-α levels in nasosinal polyps following in vitro glucocorticoid therapy. On the other hand, Hamilos et al.^15^ noted a marked decrease in GM-CSF, but not in TNF-α, in an in vivo study of fluticasone use during one month. Watanabe et al.^11^ also found decreased GM-CSF levels in nasal polyp epithelial cells under GC therapy, which would explain reduced eosinophil apoptosis.

Woodworth et al.^16^ demonstrated that IL-5 and IL-13 levels were significantly decreased in nasal polyps following systemic GC therapy in NSP patients. An even more significant reduction was observed after treatment with chemokines, eotaxin and MCP-4 (monocyte chemoattractant protein) in the same group of patients.

Silvestri et al.^17^ showed that fluticasone significantly decreased β-FGF-induced fibroblast proliferation and TNF-α-induced ICAM-1 expression. This author also observed TNF-α inhibition of eotaxin release and fibroblast inhibition within polyps due to IL-4.

Molet et al.^18^ showed that IL-11, a pro-fibrotic cytokine that stimulates fibroblast proliferation and smooth muscle hyperplasia, is increased in nasal polyps. This elevation relative to the healthy mucosa occurs both in epithelial cells and in submucosal inflammatory cells, and is directly connected with type I collagen fiber accumulation. Collagen types I and III typically accumulate in NSP, which is in line with the findings above. The same author also showed that following treatment of polyps with GC (fluticasone) during four weeks, IL-11 expression was markedly decreased. Uchida et al.^19^ demonstrated in vitro that, following treatment of polyps with fluticasone, there was suppression of mRNA expression of the basic fibroblast growth factor (bFGF) and the vascular endothelial growth factor. Yaritas et al.^20^ reported similar findings, noting decreased expression of bFGF in nasal polyps following the topical use of mometasone during four weeks in individuals with polyps.

Delbrouck et al.^21^ showed in vitro that budesonide can inhibit expression of the leukocyte migration inhibitory factor (MIF), a lymphokine that counter-regulates GC, decreasing their expression in polyp epithelial and gland cells, depending on the concentration of GC.

There are two main cell resistance mechanisms against GC:

– the ratio between GRα/GRβ receptor isoforms
– transcription factors

There are two main GS isoform: GRα and GRβ. It is known that GRα is able to bind to GC after activating the GRE, after which it is considered as the active form. GRβ has the same GRE-binding capability, but does not bind to GC; GRβ, then, is considered an endogenous inhibitor of GC. Pujols et al.^13^ and Hamilos et al.^15^ reported increased GRβ expression in NSP patients, compared with controls. Valera^10^ and Li et al.^22^ observed lower GRα levels in NSP, compared to the normal mucosa. Both Pujols et al.^13^ and Valera^10^ stated that the most important factor in resistance to GC was the GRα/GRβ isoform ratio, more important then the absolute values of each isoform.

Increased expression of transcription factors is also important in the resistance to GC; when activated, these factors are able to directly inhibit bonding between GR and GC.^23^

Valera^10^ and Takeno et al.^24^ observed an increased expression of the p65 (active) and p50 (constitutional) NFκ B fraction in nasal polyps, compared to the control mucosa. Valera^10^ showed that p65 levels decreased significantly following the use of topical budesonide for 2 months. This author also found a higher expression of p65 in NSP cases that responded less to topical budesonide, compared to those cases that responded more favorably to medical therapy.

Baraniuk et al^25^ found that the AP-1 transcription factor c-fos (active) fraction was elevated in nasosinal polyps, and that c-fos expression in polyps was decreased following oral therapy with GC. Valera,^10^ however, showed that c-fos levels were not increased compared to the control nasal mucosa.

Further knowledge about the action of GC in NSP and the development of cell resistance mechanisms against these drugs increase the possibility that more specific and effective drugs may be developed for the treatment of NSP.

## CONCLUSION

Systemic or topical corticosteroids are important drugs in the treatment of NSP, both singly in therapy and as adjuvant therapy in extensive cases that are also treated surgically. Their efficacy is still limited to 60–80% of cases.

Understanding the action mechanisms of GC in NSP may foster the development of more effective drugs for treating this condition.
